# Overexpression of tissue inhibitors of metalloproteinase 2 up-regulates NF-κB activity in melanoma cells

**DOI:** 10.1186/1750-2187-4-4

**Published:** 2009-07-23

**Authors:** Jun Sun, William G Stetler-Stevenson

**Affiliations:** 1 Department of Medicine, Department of Microbiology and Immunology, University of Rochester, 601 Elmwood Avenue, Rochester, NY 14642, USA; 2 Radiation Oncology Branch, National Cancer Institute, National Institutes of Health, Bethesda, Maryland, 20892, USA

## Abstract

**Background:**

Matrix Metalloproteinase functions in the remodeling of the extracellular matrix that is integral for many normal and pathological processes such as morphogenesis, angiogenesis, tissue repair, and tumor invasion. The tissue inhibitor of the metalloproteinase family including the tissue inhibitor of metalloproteinase-2 (TIMP-2) regulates the activity of multifunctional metalloproteinase. It is known that IL-8, the target gene of NF-κB pathway, increases in the melanoma cells. However, it is not clear whether the TIMP-2 expression regulates the NF-κB pathway. In this study, we have used stable melanoma cell lines, parental A2058, A2058T2-1 overexpressing TIMP-2, and A2058T2R-7 underexpressing TIMP-2, to determine the TIMP-2 regulation of the NF-κB activity.

**Results:**

We found that the IL-8 secretion and IL-8 mRNA expression significantly increased in the A2058T2-1 overexpressing TIMP-2. TIMP-2 overexpressed cells had the lower basal level of IκBα, the inhibitor of NF-κB, compared to the parental A2058 cells. The transcriptional NF-κB activity was increased by the TIMP-2 overexpression. In contrast, A2058T2R-7 underexpressing TIMP-2 had the similar NF-κB activity as that in the parental A2058 cell. The apoptotic cells induced by TNF were less in TIMP-2 over-expression cells compared to those in the parental A2058 cells. TIMP-2 over-expression was able to protect cells from apoptosis.

**Conclusion:**

Our data demonstrate that the expression level of TIMP-2 protein can directly modulate the NF-κB pathway in human melanoma cells.

## Background

The tissue inhibitor of the metalloproteinases family including the tissue inhibitor of metalloproteinases-2 (TIMP-2) regulates the activity of multifunctional metalloproteinases, which regulate the pathogenesis of melanoma and other diseases [1,2]. Nuclear factor-κB (NF-κB) is a family of transcription factors that play an essential role in innate and adaptive immune responses, cell proliferation, apoptosis, and tumorigenesis [3-6]. Constitutive activation of nuclear factor-B (NF-B) has been directly implicated in tumorigenesis of various cancer types, including melanoma [3-5]. NF-κB is active in the nucleus and its activity is inhibited by the inhibitor of κBα (IκBα). IκBα binds to NF-κB to block the nuclear localization signal so that the NF-κB dimer (p50 & p65) is retained in the cytoplasm. Phosphorylation of IκBα by IκB kinase (IKK) initiates the ubiquitination and degradation of IκB, leading to nuclear translocation and activation of NF-κB [6]. It is known that IL-8, the target gene of NF-κB, increases in the melanoma cells [7,8]. However, the upstream signalling pathways leading to NF-κB activity in malignant melanoma are unknown until today. It is not clear whether TIMP-2 expression directly regulates the proinflammatory NF-κB pathway.

We have established stable melanoma cell lines: parental A2058 expressing, A2058T2-1 overexpressing, and A2058T2R-7 underexpressing TIMP-2 [9]. Alternation of the TIMP-2-production is correlated with changes in the morphology of the infectant cell lines. A2058T2R-7 cells underexpressing TIMP-2 are smaller, more elongated, and spindle shaped in appearance compared to the parental A2058. A2058T2-1 overexpressing TIMP-2 cells have more sites for peripherial cell attachment and are larger, more spread than the A2058 parental cells. In addition, TIMP-2 expression has an effect on the cell attachment. Cell line with overexpression TIMP-2 showed increased adhesion to tissue culture plastics, gelatine, fibronectin, and vitronectin [9]. In the current study, we used these cell lines to examine the relationship between TIMP-2 expression and NF-κB activity in melanoma cells. The TIMP-2 regulation of the NF-κB activity was investigated at different levels including total and phosphorylated IκBα, p65 phosphorylation, NF-κB transcriptional activity, target gene IL-8 expression, and cell apoptosis.

## Results

### TIMP-2 expression increases the IL-8 protein secretion and IL-8 mRNA expression

**Figure 1:**
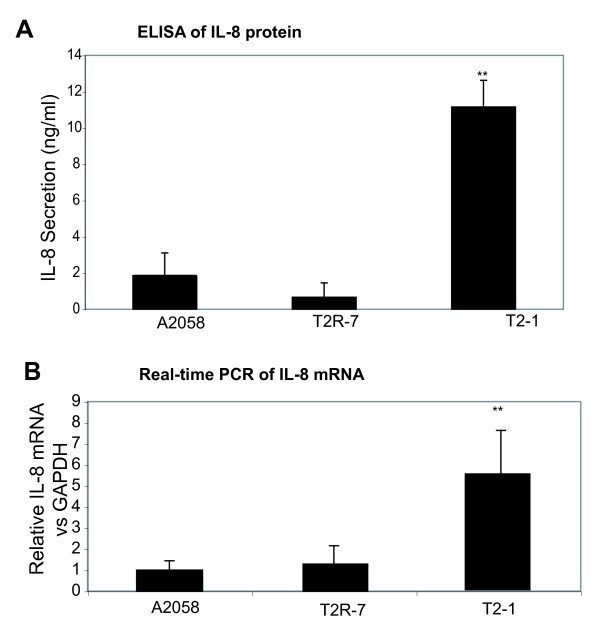
**IL-8 protein and mRNA expression in cell lines A2058, A2058T2-1, and A2058T2R-7**. (A) IL-8 secretion in cell lines A2058, A2058T2-1, and A2058T2R-7. Equal number of cells was plated in 6-well plates. After growing for 24 hours, the supernatant was collected and assayed for IL-8 ELISA. Data are the mean ± SD of a single experiment assayed in triplicate and is representative of 3 separate experiments. **P < .001 for A2058 vs A2058T2-1. (B) IL-8 mRNA expressionin the A2058, A2058T2-1, and A2058T2R-7 cells. Total RNA was extracted using TRIzol reagent and the RNA integrity was verified by electrophoresis gel. All IL-8 expression levels were normalized to the GAPDH levels of the same sample. Percent expression was calculated as the ratio of the normalized value of each sample to that of the corresponding untreated control cells. All real-time PCR reactions were performed in triplicate. **P < .001 for A2058 vs A2058T2-1.

Many pro-inflammatory cytokines and chemokines, such as IL-8, IL-6, and TNF-α, are targets of NF-κB regulation[10,11]. Because IL-8 secretion increased in the melanoma cells, we first assessed the effect of TIMP-2 expression on the IL-8 secretion in cells cultured in DMEM for 24 hours and reached 70% confluence. As shown here (Fig. 1A), there is a significant difference of IL-8 secretion in the cell lines with different level of TIMP-2 expression. TIMP-2 over-expression in A2058T2-1 cells significantly increased the IL-8 protein secreted in the cell media. In addition, IL-8 real-time PCR showed that the IL-8 mRNA was increased with TIMP-2 over-expression in A2058T2-1 cells without any stimulation (Fig. 1B).

### TIMP-2 over-expression decreases IκBα, inhibitor of NF-κB activity

**Figure 2:**
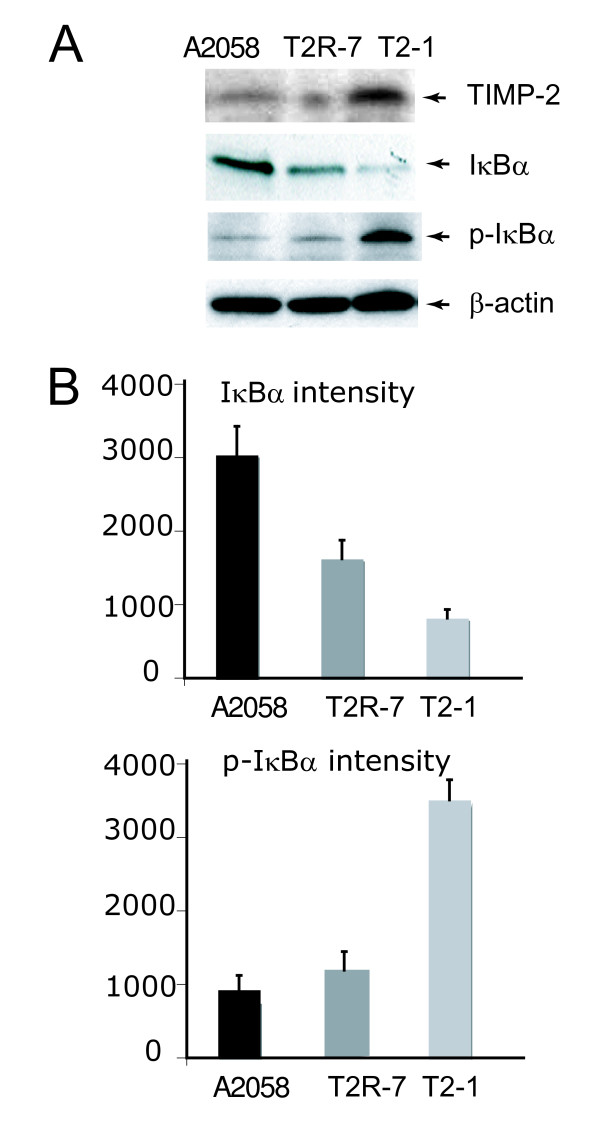
**Immunoblot of whole cell IκBα and phospho-IκBα in cell lines A2058, A2058T2-1, and A2058T2R-7**. (A) Western blot of TIMP-2, IκBα, and phospho-IκBα. (B) Densitometry of IκBα and phospho-IκBα in cell lines A2058, A2058T2-1, and A2058T2R-7. Relative band intensity was determined using NIH Image 1.63 software. Data are reported as mean ± SD of 3 independent experiments.

One assay commonly used to demonstrate the activation of the pro-inflammatory NF-κB signalling pathway is the degradation of the NF-κB inhibitory molecule IκBα (Fig. 2A). We found that the basal level of IκBα in the TIMP-2 over-expressed cells was lower than that in the parental A2058 cells. IκBα degradation involves phosphorylation, ubiquitination, and subsequent proteasomal degradation. Without any stimulation, the alteration of the phosphorylated IκBα (phosphor- IκBα) could be detected in these three cell lines. Phosphor- IκBα was increased in the TIMP-2 over-expressed cells (Fig. 2B), which is consistent with the reduced level of total IκBα.

### TIMP-2 over-expression elevates the phosphorylation of NF-κB p65

**Figure 3:**
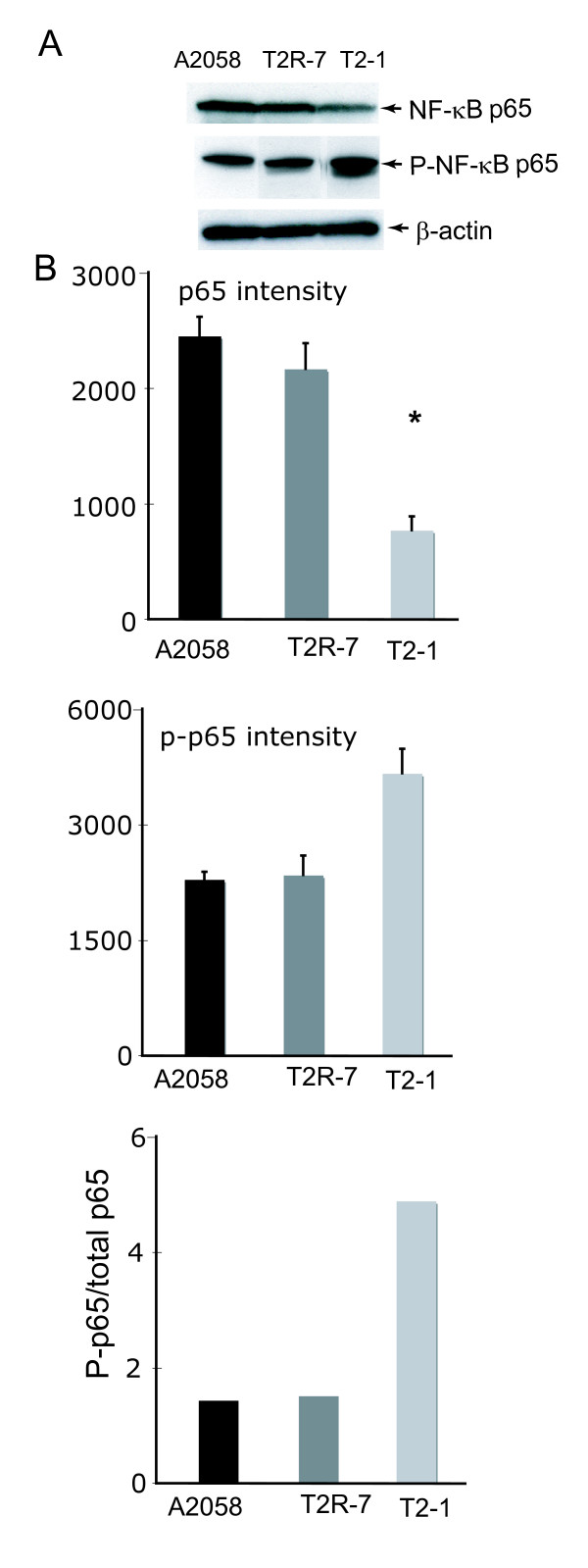
**Immunoblot of whole cell NF-κBp65 and phospho- p65 in cell lines A2058, A2058T2-1, and A2058T2R-7**. (A) Western blot of p65 and phospho-p65; (B) Densitometry of p65 and phospho-p65. Relative band intensity was determined using NIH Image 1.63 software. Data are reported as mean ± SD of 3 independent experiments.

It should be noted that NF-κB activity is regulated by phosphorylation. Transcriptional activity of NF-κB is controlled by phosphorylation of p65 at serine 536 [12,13]. The increased level of phosphorylated NF-κB P65 (p-P65) indicates the high activity of NF-κB pathway. We further determined the total p65 and p-p65 expression by Western blot (Fig. 3A). There was only a sight difference of the total p-65 expression in the three cell lines: A2058 T2-1 with overexpression of TIMP-2 had less total p65 compared to the parental cell line A2058. Interestingly, p-p65 increased in TIMP2 over-expressed A2058 T2-1 cells. The relative ratio of phosphor-p65/total p65 is the highest in the A2058 T2-1 cells compared with those in the parental cell line A2058 and A2058 T2R-7 cells (Fig. 3B). It suggests that the TIMP-2 expression is able to elevate p65 phosphorylation, thus increasing the NF-κB activity (total IκBα↓ = p-IκBα↑ = p-p65↑ = activity of NF-κB↑). This data is consistent with the increased IL-8 secretion and p-IκBα expression.

### Transcriptional activity of NF-κB is increased by TIMP-2

**Figure 4:**
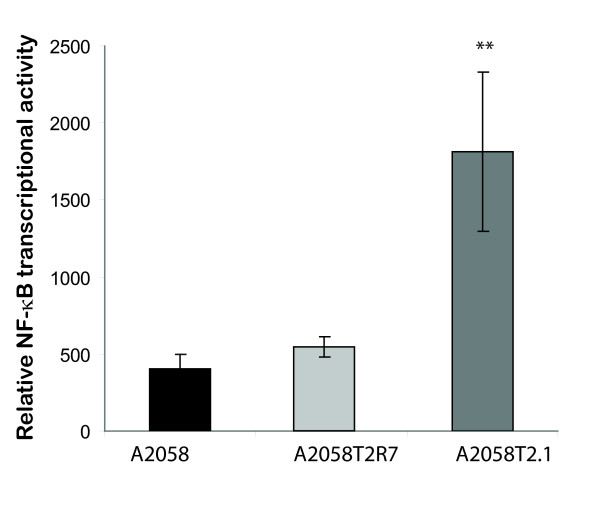
**Transcriptional activity of NF-κB in cell lines A2058, A2058T2-1, and A2058T2R-7**. NF-κB transcription was measured by luciferase activity. Data are reported as mean ± SD of a single experiment assayed in triplicate and is representative of 3 separate experiments. ** p < 0.001 for A2058 group vs A2058T2-1 or A2058T2R-7 vs A2058T2-1.

We further assessed the transcriptional activity of NF-κB by luciferase reporter assay. NF-κB activity reporter was transfected in the parental A2058, A2058T2-1, and A2058 T2R-7 cells. Without any stimulation, the level of NF-κB was significantly higher in the TIMP-2 over-expressed cells compared to that in the parental A2058 (Fig. 4). There was no significantly difference in the transcriptional activity of NF-κB between the parental A2058 and the A2058 T2R-7 cells (with underexpression of TIMP-2). It suggests that endogenous TIMP-2 expression directly up-regulates the transcriptional activity of NF-κB.

### TIMP-2 overexpression inhibits TNF-induced apoptosis

**Figure 5:**
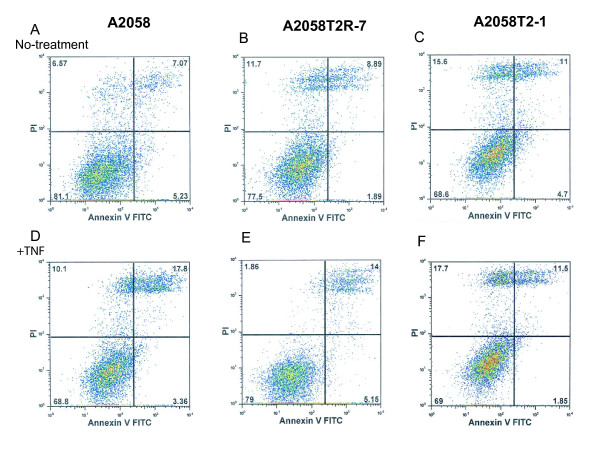
**Apoptosis in A2058, A2058T2-1, and A2058T2R-7 with or without TNF-α treatment**. After 6 h of treatment with TNF, 1 × 10^6 ^adherent cells were trypsinized and incubated with FITC-conjugated annexin V and propidium iodide (PI) for 15 min in the dark. Cells were analyzed by flow cytometry. The percentages of cells in each groups within the gated areas are indicated. In the gate areas, the up-right panel is for the population of later apoptotic cells and the low-right panel is for the early apoptosis cells. Dot plots of early apoptotic cells have increased Annexin V^FITC ^fluorescence only, whereas necrotic and late apoptotic cells have increased annexin V and PI fluorescence. Axes are labelled with arbitrary fluorescence units. Data are from a representative of three independent experiments that gave similar results.

Apoptosis is one of the biological effects regulated by the NF-κB pathway. We then determined whether the TIMP-2 expression influences cell apoptosis. As showed here, without any stimulation, the parental A2058 had about 7.07% later apoptosis/necrosis cells (Fig. 5A up-right panel), whereas TIMP-2 underexpressed A2085T2R-7 cells had about 8.89% apoptotic/necrotic cells, and TIMP-2 overexpression cells had 11% apoptotic/necrotic cells, (Fig. 5C). TNF treatment induced 17.8% (Fig. 5D) apoptosis in the parental A2058 and 14% in A2085 T2R-7 (Fig. 5E). In contrast, there were still 11% apoptotic/necrotic cells in TNF treated A2058T2-1 cells with TIMP-2 overexpression (Fig. 5F). The low-right panel in Fig. 5A represents the early apoptosis cells. After TNF treatment, the parental A2058 had about 3.36% early apoptosis/necrosis cells (Fig. 5A low-right panel), whereas TIMP-2 overexpressed A2085T2-1 cells had about 1.85% early apoptotic cells. These data indicate that TIMP-2 over-expression is able to protect cells from apoptosis and necrosis.

## Discussion

In this study, we have used stable melanoma cell lines, parental A2058, A2058T2-1 overexpressing TIMP-2, and A2058T2R-7 underexpressing TIMP-2, to determine the TIMP-2 regulation of the NF-κB activity. Our data clearly indicate the effects of TIMP-2 on the NF-κB pathway including the decreased basal level of IκBα, increased phosphorylation of IκBα and NF-κB, increased transcriptional NF-κB activity, and elevated IL-8 levels in the TIMP-2-overexpressed A2028T2-1 cells. As a biological effect regulated by the NF-κB pathway, TIMP-2 over-expression is able to protect cells from apoptosis.

Our results demonstrate that TIMP-2 expression can directly modulate the NF-κB pathway in melanoma cells. Studies in lung cancer cells demonstrated that NF-κB activity was increased by exposure to TIMP-2 as well [14]. The NF-κB transcription factor is known to act as a tumor promoter [6,15]. It is intriguing that TIMP-2 up-regulates NF-κB activity, whereas TIMP-2-overexpression can prevent tumor invasion [16]. Other data suggest a dual function of NF-κB during tumor progression [17]. In the early stages, NF-κB inhibits tumor growth; as further mutations lead to a loss of tumor suppressor expression, the oncogenic functions of NF-κB become unleashed, allowing it to actively contribute to tumorigenesis [17]. Our data indicate that elevated endogenous TIMP-2 can up-regulate the NF-κB activity, which may inhibit tumor growth in the early stage. In addition, over-expression of TIMP-2 in the cells protects cells from apoptosis induced by proinflammatory cytokine TNF. After TNF stimulation, we also measured the NF-κB transcriptional activity by the reporter luciferase assay. We found the NF-κB transcriptional activity in the TIMP-2 overexpressed cells is higher than that in the parental A2058 with TNF treatment (data not shown). However, the IL-8 secretion are equally high in there cell lines after TNF stimulation. We did not find any significant difference among these three cell line (see additional file 1). Since the NF-κB pathway regulates expression of a variety of cytokines and chemokines in addition to IL-8, changes in endogenous TIMP-2 expression may result in a change in the other proinflammatory cytokines. It will be intriguing to further determine the effects of TIMP-2 in cells under stimulation of chronic inflammation.

Previous studies demonstrate that alternation of the TIMP-2-prodcution is correlated with changes in the morphology, cell spread, and adhesion of the infectant A2058 cell lines. Over-expression of TIMP-2 blocks cell surface proteolysis required for release of cells from the matrix, as well as degradation of the extra cellular matrix, both of which would compromise the process of cellular invasion. Therefore, not surprisingly, in an *in vivo *angiogenesis assay, TIMP-2-transfected cells had reduced levels of blood vessel formation. Conditioned media from TIMP-2 transfectants diminished induction of endothelial cell migration and invasion. TIMP-2 over-expression limited tumor growth *in vivo *[16].

TIMP-2 was shown to stimulate proliferation in human cells, including osteosarcoma cells, fibroblasts, and A549 lung adenocarcinoma cells [18-20]. Our data suggest that TIMP-2 over-expression is able to protect cells from apoptosis in human melanoma A2058 cells. It is consistent with the previous studies that TIMP-2 overexpression protects B16F10 melanoma cells from apoptosis [16]. Overall, these data indicate that TIPM-2 modulates other relevant aspects of the melanomatatic phenotypes including cell proliferation and cell survival.

Previous studies showed different TIMP-2 concentration in the condition media from these three cell lines: A2058 1.4 ng/10,000 cells/24 hours, A2058T2-1 6.8 ng/10,000 cells/24 hours, and A2058 T2R-7 0.7 ng/10,000 cells/24 hours. Alteration of TIMP-2 concentration secreted in the media may contribute to the status of the NF-κB pathway and IL-8 secretion in the cell media. The major focus of current manuscript is on the cells without any stimulation. In future studies, we will investigate whether increased exogenous TIMP-2 level leads to the activation of the NF-κB activity in melanoma cells. Since the TIMP-2 under-expressed cell line is not able to completely block the protein expression of TIMP-2, a TIMP-2 knock-out cell line need be established for the investigation of TIMP-2 regulation of the NF-κB activity *in vitro *and *in vivo*.

An inflammatory tumor microenvironment fosters tumor growth, angiogenesis, and metastatic progression. Targeting NF-κB has potential therapeutic effects in clinical trials. An important step in this direction is to delineate the important intracellular pathways and upstream kinases involved in the up-regulation of NF-κB in melanoma cells. Future study will identify an inflammatory link through TIMPs and NF-κB pathway in melanoma. Understanding the role of TIMP and NF-κB interaction will allow us to elucidate how inflammation modulates tumorigenesis.

## Conclusion

In summary, our results show that TIMP-2 over-expression is sufficient to increase the NF-κB activity and protect cells from apoptosis. To our knowledge, this is the first report on the TIMP-2 upregulation of NF-κB activity in melanoma cells. Our data emphasize the critical role of TIMP-2 in modulating cell survival and invasion through the NF-κB activity.

## Methods

### Cell culture

Human melanoma A2058, A2058T2-1, and A2058T2R-7 cells were maintained in DMEM supplemented with 10% FBS and penicillin-streptomycin as previously described [9]. Equal number of cells was plated in 6-well plates. After growing for 24 hours, cells were harvested for assays.

### Immunoblotting

Cells were plated in 6-well plates. After growing for 24 hours and reaching about 70%–80% confluence, cultured cells were rinsed twice in ice-cold HBSS, lysed in protein loading buffer (50 mM Tris, pH 6.8, 100 mM dithiothreitol, 2% SDS, 0.1% bromphenol blue, 10% glycerol), and sonicated. Equal amount of proteins or equal volumes of total cultured cell lysates were separated by SDS-polyacrylamide gel electrophoresis, transferred to nitrocellulose, and immunoblotted with primary antibodies (1: 500 to 1000 dilution): anti-phospho-IκBα (Ser^32/36^) antibody (Cell Signaling Technology, Danvers, MA, USA), anti-phospho-p65 on serine 536 (Cell Signaling Technology), anti-IκBα, anti-p65 (Santa Cruz Biotechnology, Santa Cruz, CA, USA), or β-actin (Sigma-Aldrich, St. Louis, MO, USA) antibodies and visualized by ECL as previously described [21,22].

### IL-8 secretion

Equal number of cells was plated in 6-well plates. After growing for 24 hours, the supernatant was collected and assayed for IL-8 using the R&D Systems human IL-8 ELISA kit (R&D, Inc., Minneapolis, MN, USA) according to the manufacturer's instructions as previously described [21-23].

### Quantitative real-time PCR analysis

Total RNA was extracted from epithelial cell monolayers using TRIzol reagent (Invitrogen, Carlsbad, CA). The RNA integrity was verified by electrophoresis gel. RNA reverse transcription was done using the iScript cDNA synthesis kit (Bio-Rad, Hercules, CA) according to the manufacturer's directions. The RT cDNA reaction products were subjected to quantitative real-time PCR using the MyiQ single-color real-time PCR detection system (Bio-Rad) and iQ SYBR green supermix (Bio-Rad) according to the manufacturer's directions. IL-8 cDNA was amplified by using primers to the human IL-8 gene that are complementary to regions in exon 1 (5'-TGCATAAAGACATACTCCAAACCT) and overlapping the splice site between exons 3 and 4 (5'-AATTCTCAGCCCTCTTCAAAAA). All expression levels were normalized to the GAPDH levels of the same sample, using forward (5-CTTCACCACCATGGAGAAGGC) and reverse (5'-GGCATGGACTGTGGTCATGAG) primers for GAPDH. Percent expression was calculated as the ratio of the normalized value of each sample to that of the corresponding untreated control cells. All real-time PCR reactions were performed in triplicate. All PCR primers were designed using Lasergene software (DNAStar, Madison, WI).

### Cell transfection

Cells were grown in 24-well plates in triplicates. At ~70–80% confluence, the cells were cotransfected with NF-κB reporter plasmid pNF-κB-Luc (Stratagene, La Jolla, CA, USA) and control plasmid pRL-TK (Promega, Madison, WI, USA) using LipofectAMINE (Invitrogen, Carlsbad, CA, USA). After 24 h, the cells were lysed, and luciferase activity was determined using the Dual Luciferase Reporter Assay System (Promega). Firefly luciferase activity was normalized with *Renilla *luciferase activity, and the activity was expressed as relative units as previously described [21,22].

### Apoptosis assays

After 6 h of treatment with TNF, 1 × 10^6 ^adherent cells were trypsinized and incubated with FITC-conjugated annexin V (binds to phosphatidyl serine on the cytoplasmic surface of the cell membrane) and propidium iodide (PI) for 15 min in the dark according to the manufacturer's protocol (Annexin V^FITC ^Apoptosis Detection kit; Oncogene Research Products, San Diego, CA, USA). Cells were analyzed by flow cytometry.

## Competing interests

The authors declare that they have no competing interests.

## Authors' contributions

JS conceived the project and developed the conception of the study, designed and coordinated the study, performed the experiment, and prepared manuscript. WGS provided the cell lines, participated in the conception of the study and interpretation of the data, and helped to prepare the manuscript. All authors read and approved the final manuscript.

## Supplementary Material

Additional file 1 **IL-8 protein secretion in cells stimulated with TNF**. The data provided IL-8 protein levels in unstimulated cells and cells stimulated with TNF for 6 hours. Click here for file
