# Supplementary material for: Overexpression of tissue inhibitors of metalloproteinase 2 up-regulates NF-κB activity in melanoma cells
Source: J Mol Signal. 2009 Jul 23;4:4. doi: 10.1186/1750-2187-4-4 (PMC2720935; doi:10.1186/1750-2187-4-4)
Supplement: Additional file 1 — IL-8 protein secretion in cells stimulated with TNF. The data provided IL-8 protein levels in unstimulated cells and cells stimulated with TNF for 6 hours. [file 1750-2187-4-4-S1.pdf]

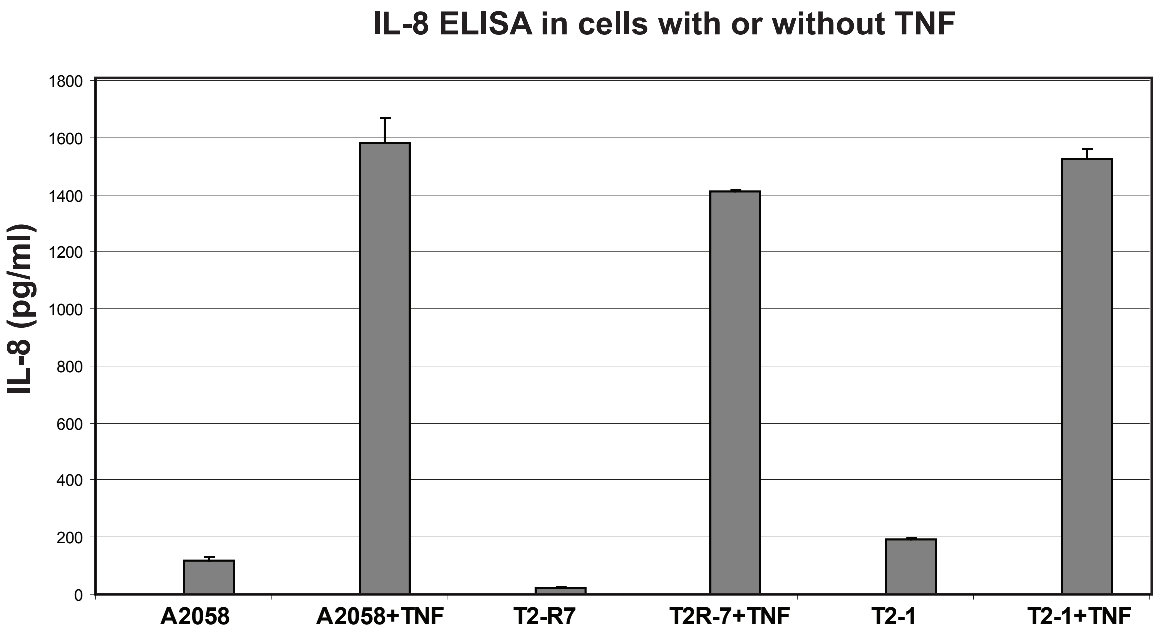

Supplement data figure 1. Equal number of cells was plated in 6-well plates. After growing for 24 hours, cells were stimulated with TNF for 6 hours. The supernatant was collected and assayed for IL-8 using the R&D Systems human IL-8 ELISA.
